# Economic evaluation of a weight control program with e-mail and telephone counseling among overweight employees: a randomized controlled trial

**DOI:** 10.1186/1479-5868-9-112

**Published:** 2012-09-11

**Authors:** Marieke F van Wier, J Caroline Dekkers, Judith E Bosmans, Martijn W Heymans, Ingrid JM Hendriksen, Nicolaas P Pronk, Willem van Mechelen, Maurits W van Tulder

**Affiliations:** 1 Department of Public and Occupational Health, VU University Medical Center, Amsterdam, The Netherlands; 2 Department of Epidemiology and Biostatistics, VU University Medical Center, Amsterdam, The Netherlands; 3 EMGO Institute for Health and Care Research, VU University and VU University Medical Center, Amsterdam, The Netherlands; 4 Body@Work, Research Center Physical Activity, Work and Health, TNO-VUmc, Amsterdam, The Netherlands; 5 Municipal Health Service, The Hague, The Netherlands; 6 Institute of Health Sciences, Faculty of Earth and Life Sciences, VU University, Amsterdam, The Netherlands; 7 TNO Quality of Life, Leiden, The Netherlands; 8 HealthPartners, JourneyWell and HealthPartners Research Foundation, Minneapolis, MN, USA

**Keywords:** Body weight, Cost-effectiveness, Cost-utility, Distance counseling, Intervention, Lifestyle, RCT, Workplace health promotion

## Abstract

**Background:**

Distance lifestyle counseling for weight control is a promising public health intervention in the work setting. Information about the cost-effectiveness of such interventions is lacking, but necessary to make informed implementation decisions. The purpose of this study was to perform an economic evaluation of a six-month program with lifestyle counseling aimed at weight reduction in an overweight working population with a two-year time horizon from a societal perspective.

**Methods:**

A randomized controlled trial comparing a program with two modes of intervention delivery against self-help. 1386 Employees from seven companies participated (67% male, mean age 43 (SD 8.6) years, mean BMI 29.6 (SD 3.5) kg/m^2^). All groups received self-directed lifestyle brochures. The two intervention groups additionally received a workbook-based program with phone counseling (phone; n=462) or a web-based program with e-mail counseling (internet; n=464). Body weight was measured at baseline and 24 months after baseline. Quality of life (EuroQol-5D) was assessed at baseline, 6, 12, 18 and 24 months after baseline. Resource use was measured with six-monthly diaries and valued with Dutch standard costs. Missing data were multiply imputed. Uncertainty around differences in costs and incremental cost-effectiveness ratios was estimated by applying non-parametric bootstrapping techniques and graphically plotting the results in cost-effectiveness planes and cost-effectiveness acceptability curves.

**Results:**

At two years the incremental cost-effectiveness ratio was €1009/kg weight loss in the phone group and €16/kg weight loss in the internet group. The cost-utility analysis resulted in €245,243/quality adjusted life year (QALY) and €1337/QALY, respectively. The results from a complete-case analysis were slightly more favorable. However, there was considerable uncertainty around all outcomes.

**Conclusions:**

Neither intervention mode was proven to be cost-effective compared to self-help.

**Trial registration:**

ISRCTN04265725

## Background

Globally the number of people who are overweight, defined as having a Body Mass Index (BMI) of 25 kg/m^2^ or higher, is increasing. In the Netherlands, almost half of the population is overweight [1]. Overweight is linked to the development of chronic diseases like type 2 diabetes, cardiovascular disease and certain types of cancer and has a considerable impact on public health [2]. The increased prevalence of overweight also has an impact on the work setting. In comparison with employees with a BMI below 25 kg/m^2^, overweight employees have longer sick leave spells [3] and are at increased risk for work disability [4]. Treating and preventing overweight among employees could result in health gains and possible cost reductions due to decreased health care use and absenteeism. Implementation of weight control programs in the occupational health care setting may be advantageous from both a company and a societal perspective. However, economic evaluations of interventions are needed to guide implementation decisions.

The motivation behind health economic evaluations is getting the most benefit from the scarce resources available to society. Economic evaluations should therefore take a societal perspective.[5] This societal perspective implies inclusion of all relevant costs and effects, regardless of who pays the costs or who receives the benefits [6]. The societal costs are weighed against health benefits. An advantage of the societal perspective over narrower perspectives is that it shows the distribution of costs and benefits over societal payers and allows for bargaining between them [5]. In the Netherlands, companies pay for occupational health care and prevention. They have to make decisions within a tight budget regarding allocation of resources. Therefore, the narrower perspective of the company, weighing the costs and benefits to employers, may also be relevant in economic evaluations of workplace health promotion.

Interventions for weight control in the clinical setting are usually based on behavior modification and comprise several face-to-face meetings, either individually or in a group. Several modeling studies have shown that these interventions may be cost-effective from a societal perspective [7]. Yet, face-to-face interventions could be impractical in the work setting. Employees mention constraints of time and location as barriers for participation in them [8]. Programs that make use of distance communication technology for person-to-person counseling, like e-mail and telephone, have the potential to be more accessible to employees. Limited evidence is available of the cost-effectiveness of these methods in addressing body weight and weight-related behaviors. Economic evaluations in healthy working-age adults concluded superior cost-effectiveness for a mix of e-mail and phone counseling [9-11] and inferior cost-effectiveness for phone counseling alone [12,13], compared with usual care, an alternative intervention or no intervention. The purpose of this study was to investigate the cost-effectiveness for weight reduction and cost-utility of a lifestyle program utilizing e-mail or phone counseling in comparison with self-help among overweight employees, from a societal perspective and with a time horizon of two years.

## Methods

### Study design

An economic evaluation was conducted alongside a randomized controlled trial (RCT) with three study-arms, carried out in the Netherlands from 2004 to 2007. Details of the study design, the intervention and its effectiveness on body weight and cardiovascular risk factors after six months and two years have been published before [14-17]. The study was approved by the Medical Ethics Committee of the VU University Medical Center and all participants provided written informed consent. The trial has been registered at isrctn.org as ISRCTN04265725.

### Participants and setting

Seven different service-sector companies in the Netherlands participated in this study. Employees of these companies were eligible if they met the following criteria: BMI ≥ 25 kg/m^2^, paid employment for at least 8 hours a week, able to read and write Dutch, access to and making regular use of the internet, age 18 years and older, not pregnant and no diagnosis or treatment for disorders that would make physical activity difficult (for example knee osteoarthritis). Employees who were willing to participate were randomized using a blinded allocation schedule. The participants and counselors were, in consequence of the nature of the intervention, not blinded for the intervention.

### Interventions

According to two reviews from 2006, information on diet and physical activity is only incidentally given in the Dutch occupational healthcare setting [18,19]. Thus, usual occupational care for overweight employees likely consists of no care at all. However, having a no-care group was thought to hamper recruitment to the study. For that reason, all groups including the control group, received self-help brochures on lifestyle change. Additionally, participants in the two intervention groups received a lifestyle intervention program consisting of ten modules [14]. These modules gave information on nutrition and physical activity, and taught behavior modification strategies (e.g. self-monitoring, goal setting). After finishing each module, participants were contacted by their personal counselor. The phone group received the program in written form and was contacted by phone. The internet group had access to an interactive and individualized program website and was counseled by e-mail. Participants in the internet group received automated twice-weekly e-mails to encourage them to start and finish modules. Counselors made an appointment with participants in the phone group for the next phone session. If a participant could not be reached at the set date and time, one more phone call was made. If this was unsuccessful, an e-mail was sent asking the participant to contact the counselor. Counseling was provided for a period of six months and discontinued if the participant declined further contact. A step counter was given to the phone and internet group, as a motivational aid for increasing physical activity.

### Study measures

Measurements consisted of a mixture of physical measurements and questionnaires, as explained below. Research-related follow-up, including follow-up of participants who discontinued their allocated intervention, was pursued with up to five reminders by mail, e-mail and telephone.

### Health outcomes

The primary outcome of the study was change in body weight from baseline to 24 months. Baseline and 24-month follow-up body weight measurements were done at or near the workplace [14]. Body weight was measured using a digital scale (Seca 770; Seca GmbH & Co, Hamburg, Germany) with participants wearing light clothes and no shoes. Body weight was also measured at 6 months, and self-reported body weight was collected by questionnaire at baseline, 6, 12, 18 and 24 months. Current body weight was asked from participants who decided to withdraw from the study. When weight measurements at the 24-month follow-up were missing, but self-reported weight at baseline and 24-month follow-up (± 3 months) were available, these were used in the analyses.

The EuroQol-5D (EQ-5D) was used to assess quality of life at baseline, and at 6, 12, and 24 month follow-up [20]. Health utilities were estimated with the Dutch tariff [21]. Quality adjusted life years (QALYs) were calculated by the area under the curve method. Utilities were multiplied with the amount of time a patient spent in a particular health state. Transitions between health states were linearly interpolated.

### Costs

Information on medical resource use, medication use and sickness absenteeism from paid work was obtained through prospective 6-month diaries provided to the participants at baseline, and at the 6, 12 and 18 month follow-up. Participants were asked to keep this diary for the next six months and to fill in frequency of use of each cost category per month. If no use was made of a cost category, the answer box could be left empty.

As recommended in Dutch guidelines, standard costs were used to value health care utilization such as costs of general practitioner care, allied health care, medical specialist care, complementary medicine and hospitalization [22]. When these were not available, prices reported by professional associations were used. The costs of drugs were estimated on the basis of prices charged by the Royal Dutch Society for Pharmacy [23]. Costs of production losses based on self-reported sick leave from work were estimated with the friction cost approach (friction period 154 calendar days and an elasticity of 0.8), using the mean income of the Dutch population according to age and gender [22]. Cost categories and prices used in the economic evaluation are given in Table 1. Prices were adjusted for the year 2004, the first year of measurement, using consumer price indices [24].

**Table 1:** Price weights used for valuation of resource use, per visit unless otherwise mentioned

**Type of utilization**	**Price weight**^**a**^
** Health care**	
***Intervention***	
Counseling (minute)	1.14
***Primary care***	
General practitioner	20.44^b, c^
Occupational physician	21.50
Physical therapist	23.02
Dietitian	30.12
Dentist	17.47
Complementary therapists	23.51 – 63.95^d^
Other primary care	23.02 – 77.51^b, d^
***Secondary care***	
Outpatient	56.66
Admission general hospital (day)	340.99
** Production losses**	
Sick leave (hour)	20.31 – 48.39^b, e^

^a^ Euros, corrected to the year 2004. ^b^ Dutch standard costs[22]^c^ Price for consultation at the practice; ^d^ Range of price weights for different therapists, obtained from professional organizations; ^e^ Range of possible price weights for sick leave, depending on age and sex.

Costs for the self-help materials provided to all groups were not included, as these were similar in each group. Intervention costs were based on charges paid during the development and implementation of the intervention. Interventions costs consisted of fixed (annual) costs and of counseling costs that varied per participant. The fixed costs covered costs of the development of materials and the website, printing costs, step counter costs and costs for maintaining a counseling center. Total fixed costs per participant of the phone intervention were €69 and of the internet intervention €65. During implementation of the intervention, counselors recorded the time they spent on counseling, attempts to contact the participant for counseling and administrative activities for each contact. Based on these records, counseling costs per participant were computed. Total intervention costs per participant were estimated by adding the fixed costs and counseling costs. A detailed description of the costing of the intervention can be found in Additional file 1.

Research-related costs were excluded from the cost calculations.

### Analyses

Intention-to-treat analyses were conducted based on group allocation, regardless of actual intervention received or adherence to the intervention. However, participants who died or became pregnant during the study were excluded from all analyses. In the main analyses, missing total direct costs, indirect costs, body weight and health utilities, were multiply imputed. Five different data sets were created with the Multivariate Imputation by Chained Equations procedure [25]. Group allocation, age, sex, educational level, baseline weight, available body weight at 6, 12 and 18 months (collected by questionnaires) and 24-month follow-up weight, intervention costs, and available direct and indirect costs at 6, 12, 18 and 24 months were included in the imputation model. The five data sets were analyzed separately. The estimates were then pooled using a formula described by Rubin [25]. This method does not allow for an estimation of standard deviations, so the standard error of the mean (SEM) is presented in the tables.

Regression analysis was used to compare differences in follow-up body weight between groups (i.e. phone vs. control and internet vs. control), while adjusting for baseline weight. Two-sided T-tests were used to compare QALYs gained.

To compare costs between groups, confidence intervals around the mean differences in costs were estimated using the bias-corrected and accelerated bootstrap method (BCA) with 2000 replications. Incremental cost-effectiveness ratios (ICER) and incremental cost-utility ratios (ICUR) were estimated by dividing the difference in total costs between the treatment groups by the difference in outcomes at 24 months. To graphically present uncertainty around the ratios, bootstrapped cost-effect pairs (2000 replications) were plotted on cost-effectiveness planes (CE planes) [6]. Cost-effectiveness acceptability curves (CEACs) were used to present the probability that each of the interventions is more cost-effective than the others for a range of willingness-to-pay thresholds [26]. The willingness-to-pay threshold represents the maximum amount of money a decision maker is willing to spend to obtain a unit of health outcome (e.g. QALYs). The Netherlands lack a formal threshold for societal cost-per-QALY [27]. For the current study a threshold of €20,000/QALY is applied, in line with a review of preventive interventions in the Netherlands [28].

Four sensitivity analyses were conducted to test the robustness of the results. In the first sensitivity analysis costs for the second year were discounted with 4% and QALYs achieved in this year were discounted with 1.5%, according to Dutch guidelines [29]. The second sensitivity analysis was restricted to participants with complete cost and effect data, i.e. complete case analysis. The third sensitivity analysis was done from the perspective of a Dutch company. The costs concern those that the company pays, i.e. intervention costs and absenteeism costs. Since employers want interventions that are cost-saving, the willingness-to-pay threshold is €0 for all health effects [30]. In the fourth sensitivity analysis QALYs were estimated using the UK EQ-5D tariff [31].

The statistical significance level was set at 5%, meaning that if a 95% confidence interval does not include the value of no difference, statistical significance is present.[32] Analyses were performed with SPSS version 15.0 and R version 2.7.1 [33]. CEACs were constructed using MS Excel 2007.

## Results

### Participant flow and baseline characteristics

**Figure 1:**
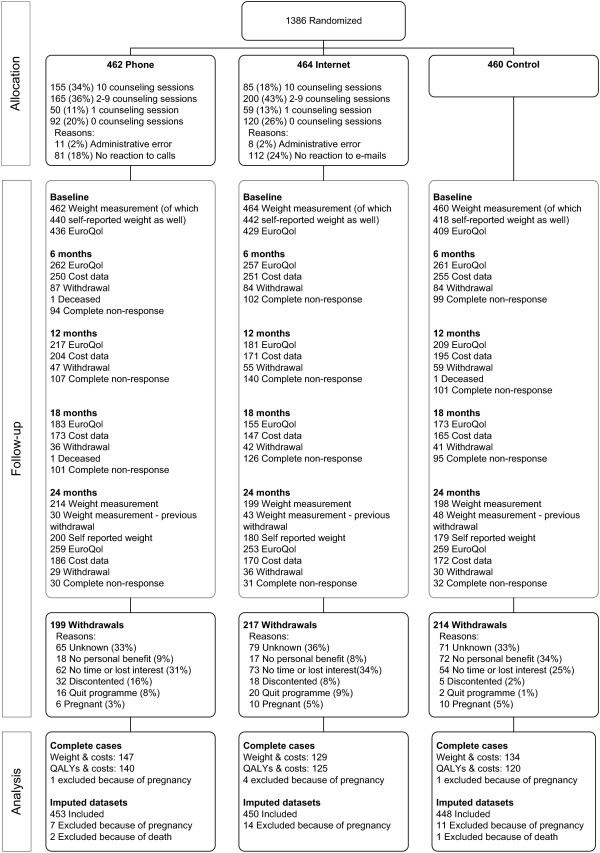
**Participant flow after randomization**^**a**^**.**^a^ The participant flow up to randomization can be found in Van Wier et al.(2009) [15]. ^b^ Costs and Quality Adjusted Life Years (QALYs) are complete when cost data and EQ5D-questionnaire are available at each measurement. Participants were approached at each measurement, unless they had dropped out from the study. Participants showed intermittent non-response (e.g. providing data at baseline, 12 and 24 months but not at 6 and 18 months,) and also partial non-response (e.g. providing complete body weight data, but not complete cost data). The number of participants with complete data therefore cannot be calculated from this participant flow.

The participant flow of the 1386 employees randomized to the phone group (N=462), internet group (N=464) and control group (N=460) is presented in Figure 1. A total of 630 participants (45%) dropped out from the study and three participants died of unknown causes. Lack of time or loss of interest in the study and, for the control group, lack of personal benefit, were mostly given as reason for leaving the study (Figure 1). To increase the follow-up rate, dropouts (except those that dropped out because of pregnancy or disappointment in the study) were approached and asked if they were willing to attend the 24-month measurements. Out of the 549 approached, 121 were willing to do so.

Because utilities and costs had to be available at all measurement times to be complete and most participants missed at least one measurement, body weight and costs were complete for 410 (30%) participants. Utilities and costs were complete for 385 (28%) participants. For the main analysis, 43% of follow-up body weight, 41% of health utilities, and 57% of cost data were imputed.

Baseline characteristics of all randomized participants are given in Table 2. Over half of the participants were male, mean age was 43 years and mean BMI was 29.6 kg/m^2^.

**Table 2:** Baseline characteristics of the control, phone and internet group, and of all participants

	**Control n=460**	**Phone n=462**	**Internet n=464**	**All n=1386**
Male, n (%)	306 (66.5)	321 (69.5)	302 (65.1)	929 (67.0)
Age (years)	43.2 (8.7)	43.2 (8.8)	43.4 (8.4)	43.3 (8.6)
Body weight (kg)	92.8 (13.6)	93.3 (14.1)	92.7 (14.3)	92.9 (14.0)
Body Mass Index (kg/m^2^)	29.6 (3.7)	29.5 (3.5)	29.6 (3.4)	29.6 (3.5)
Health utility^a, b^	0.908 (0.136)	0.917 (0.129)	0.915 (0.117)	0.913 (0.128)
Sick leave in previous 3 months (days) ^c^	1.9 (6.0)	3.4 (11.1)	2.6 (9.6)	2.7 (9.2)
0 days, n (%)	267 (63.1)	291 (62.5)	315 (70.6)	873 (66.4)
1 – 7 days, n (%)	130 (30.7)	114 (25.6)	95 (21.3)	339 (25.8)
8 – 30 days, n (%)	24 (5.7)	28 (6.3)	28 (6.3)	80 (6.1)
> 30 days, n (%)	2 (0.5)	13 (2.9)	8 (1.8)	23 (1.7)

Values are mean (SD), unless otherwise mentioned.

^a^ n=1261; ^b^ Health utilities are expressed on a scale from 0 (death) to 1 (perfect health); ^c^ n=1315.

Loss to follow-up (i.e. missing data due to discontinuation and non-response) was equal in each study group. However, participants with missing data had a 3.4 kg higher baseline body weight (94.0 vs. 90.5 kg, 95% CI 1.9 to 4.9; results not tabulated). For those participants with missing cost data but available follow-up weight, a 2.9 kg higher two-year follow-up weight (91.8 vs. 89.0 kg, 95% CI 1.0 to 4.7) was observed compared with participants with full data. Furthermore, participants with missing data completed less counseling sessions. Participants in the phone group who had missing data completed 5.1 counseling sessions, while participants with complete data had 8.4 sessions (3.3; 95% CI 2.4 to 4.1). In the internet group this was 3.2 and 7.5 sessions respectively (4.3; 95% CI 3.5 to 5.1).

### Use of the interventions

The mean (SD) use of the interventions was 5.1 (4.2) counseling sessions in the phone group and 4.1 (3.8) sessions in the internet group. Average total counseling time was 116 (91) minutes in the phone group and 99 (99) minutes in the internet group. Of the participants in the phone group, 34% completed all sessions, compared to 18% in the internet group (Figure 1).

### Outcomes

The main analysis showed no significant differences in change in body weight between the intervention groups and control group. Mean QALYs achieved over two years were similar in each group (Table 3).

**Table 3:** **Pooled outcomes for body weight and QALYs**^**a**^**achieved between baseline and two year follow-up**

**Clinical outcome**	**Control n=448**	**Phone n=453**		**Internet n=450**	
	**Mean (SEM)**	**Mean (SEM)**	**ΔE (95% CI)**^**b**^	**Mean (SEM)**	**ΔE (95% CI)**
Weight loss (kg)	1.1 (0.33)	1.5 (0.29)	0.3 (-0.6; 1.3)	1.9 (0.27)	0.9 (-0.1; 1.9)
QALYs achieved^c^	1.85 (0.008)	1.85 (0.011)	0.001 (-0.03; 0.03)	1.86 (0.009)	0.01 (-0.01; 0.04)

^a^ QALY, Quality Adjusted Life Year; ^b^ ΔE, mean difference in clinical outcome; ^c^ The maximum amount of QALYs that can be achieved in two years is 2.0.

### Costs

Table 4 presents the mean two-year costs of each group and the mean incremental costs of the intervention groups in each main cost-category. Mean costs for the intervention were €201 for the phone-version and €177 for the internet version. There were no statistically significant cost differences between the groups, except for higher healthcare costs in the internet group compared with the control group.

**Table 4:** Pooled costs and cost differences in Euros between baseline and two year follow-up

	**Control n=448**	**Phone n=453**		**Internet n=450**	
	**Mean (SEM)**	**Mean (SEM)**	**ΔC (95% CI)**^**a**^	**Mean (SEM)**	**ΔC (95% CI)**
Intervention	0	201 (5)	201 (NA^b^)	177 (5)	177 (NA)
Health care	656 (46)	739 (61)	83 (−56; 219)	819 (90)	163 (10; 344)
Sick leave	1824 (249)	1893 (296)	69 (−731; 765)	1498 (305)	−326 (−1019; 419)
Total	2480 (273)	2832 (295)	352 (−462; 1095)	2494 (360)	14 (−790; 817)

^a^ ΔC, mean difference in total costs; ^b^ NA, not applicable.

### Cost-effectiveness for weight loss

**Figure 2:**
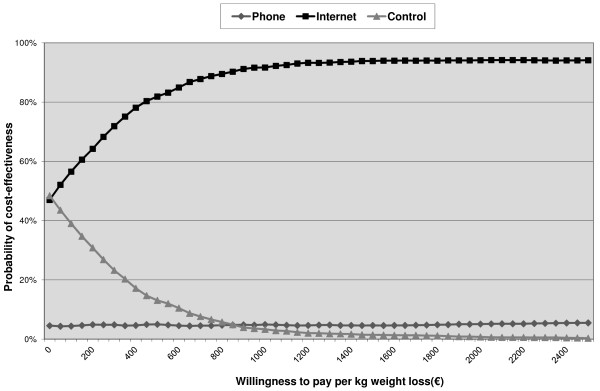
Cost-effectiveness acceptability curves for weight loss from self help (control) and two lifestyle programs with counseling by phone (phone) or e-mail (internet).

Mean incremental societal costs, incremental effects, ICERs and the distribution of cost-effectiveness pairs in the cost-effectiveness planes for the phone group are presented in Table 5 and for the internet group in Table 6. The ICERs suggest that the interventions were more effective than self help, but also more costly. The ICER for weight loss in the phone group compared with the control group was €1009 per kg weight loss, whereas it was €16 per kg weight loss in the internet group compared with the control group. The CE-planes are shown in Additional file 2. At a societal willingness-to-pay (WTP) of €0/kg, self help and the internet intervention had an equal probability of cost-effectiveness, but at higher WTP values the probability increased for the internet intervention and decreased for self help (Figure 2). The probability that the phone intervention was more cost-effective was below 5%, regardless of WTP.

**Table 5:** Incremental cost-effectiveness ratios and distribution of the joint cost-effect pairs in the cost-effectiveness planes of the phone group resulting from the main analyses and the sensitivity analyses

** Analysis**^**a**^	**Sample size per group**		**ΔC (95% CI)**	**ΔE (95% CI)**		**Distribution in CE plane (%)**			
	**Control**	**Phone**	**Euros**	**Weight loss (kg)**	**ICER**	**NE**^**b**^	**SE**^**c**^	**SW**^**d**^	**NW**^**e**^
Main	448	453	352 (−462; 1095)	0.3 (−0.6; 1.3)	1009	65	14	6	16
Complete cases	134	147	593 (−157; 1458)	1.1 (−0.02; 2.2)	543	91	7	0	2
Company perspective	448	453	270 (−525; 997)	0.3 (−0.6; 1.3)	772	62	17	7	13
				**QALY**	**ICUR**				
Main	448	453	352 (−490; 1099)	0.001 (−0.03; 0.03)	245,242	41	14	5	40
Complete cases	120	140	423 (−458; 1250)	0.006 (−0.04; 0.05)	131,863	50	13	3	34
Company perspective	448	453	270 (−525; 997	0.001 (−0.03; 0.03)	187,545	37	17	8	38
UK tariff	448	453	352 (−490; 1099)	0.007 (−0.04;0.05)	52,496	50	13	7	30

^a^ In the analysis ΔC= mean difference in total costs, ΔE= mean difference in outcome, ICER (ICUR) =incremental cost-effectiveness (utility) ratio calculated as ΔC/ΔE. In the main analysis missing data were multiply imputed. The complete cases analysis was restricted to participants with complete cost and effect data. ^b^ Northeast quadrant of the CE-plane: the intervention is more effective and more costly than self-help brochures. ^c^ Southeast quadrant of the CE-plane: the intervention is more effective and less costly than self-help brochures. ^d^ Southwest quadrant of the CE-plane: the intervention is less effective and less costly than self-help brochures. ^e^ Northwest quadrant of the CE-plane: the intervention is less effective and more costly than self-help brochures.

**Table 6:** Incremental cost-effectiveness ratios and distribution of the joint cost-effect pairs in the cost-effectiveness planes of the internet group resulting from the main analyses and the sensitivity analyses

** Analysis**	**Sample size per group**		**ΔC (95% CI)**	**ΔE (95% CI)**		**Distribution in CE plane (%)**			
	**Control**	**Internet**		**Weight loss (kg)**	**ICER**	**NE**^**b**^	**SE**^**c**^	**SW**^**d**^	**NW**^**e**^
Main	448	450	14 (−790; 867)	0.9 (−0.1; 1.9)	16	50	48	1	1
Complete cases	134	129	-82 (-838 to 633)	1.3* (0.3; 2.4)	−62	41	58	0	0
Company perspective	448	450	−149 (−858; 618)	0.9 (−0.1; 1.9)	−171	33	65	2	1
				**QALY**	**ICUR**				
Main	448	450	14 (−774; 887)	0.01 (−0.01; 0.04)	1337	35	47	5	14
Complete cases	120	125	−307 (−1179; 315)	0.02 (−0.02; 0.06)	−27,908	17	71	8	5
Company perspective	448	450	−149 (−858; 618)	0.01 (−0.01; 0.04)	−14,181	23	58	8	11
UK tariff	448	450	14 (−774; 887)	0.02 (−0.02;0.06)	702	41	47	4	9

^a^ In the analysis ΔC= mean difference in total costs, ΔE= mean difference in outcome, ICER (ICUR) =incremental cost-effectiveness (utility) ratio calculated as ΔC/ΔE. In the main analysis missing data were multiply imputed. The complete cases analysis was restricted to participants with complete cost and effect data. ^b^ Northeast quadrant of the CE-plane: the intervention is more effective and more costly than self-help brochures. Southeast quadrant of the CE-plane: the intervention is more effective and less costly than self-help brochures. ^d^ Southwest quadrant of the CE-plane: the intervention is less effective and less costly than self-help brochures. ^e^ Northwest quadrant of the CE-plane: the intervention is less effective and more costly than self-help brochures. *p=0.01.

### Cost-utility

**Figure 3:**
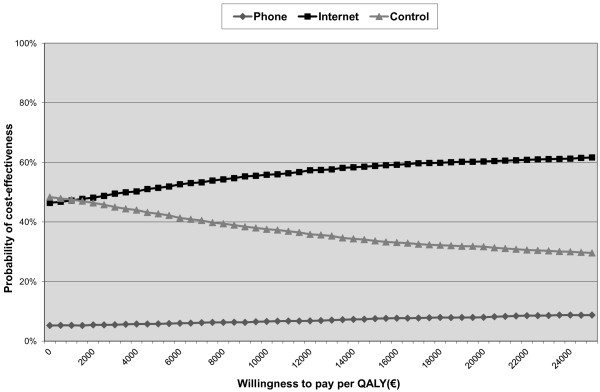
Cost-effectiveness acceptability curves for QALYs gained from self help (control) and two lifestyle programs with counseling by phone (phone) or e-mail (internet).

The ICUR of the phone group compared with the control group was €245,243 per QALY (Table 5). For the internet group compared with the control group the ICUR was €1337 per QALY (Table 6). Both ICURs implied higher effectiveness at greater costs. The CE-planes are displayed in Additional file 2, with the distribution of the cost-effectiveness pairs given in Tables 5 and 6. Cost-utility probabilities at a WTP of €20,000/QALY were 8% for the phone intervention, 60% for the internet intervention and 32% for self help (Figure 3).

### Sensitivity analyses

Results from the sensitivity analysis with discounted data were comparable with the results from the main analysis (results not shown). However, results from the complete case analysis, as presented in Table 5 for the phone group and in Table 6 for the internet group, differed from those found in the main analysis, most notably so in the internet group. Compared with self help, the internet intervention resulted in significant weight loss while societal costs were (non-significantly) lower (Table 6). An ICER of €-62 (i.e. a reduction in societal costs of €62 for each kg lost) and an ICUR of €-27,908 (i.e. a reduction in societal costs of €27,908 for each QALY gained), as compared with self help, were found (Table 6). The probability that the internet intervention was cost-effective at a WTP of €0/kg weight loss was 57% and reached a maximum of 89% at a WTP of €550. The probability of its cost-utility was 86% at €20,000/QALY.

Results from the analysis from the perspective of a Dutch company were similar to the main analysis for the phone group (Table 5). Results of the internet group showed a saving of €149 Euros (Table 6). At a WTP of €0 per unit of health effect, the likelihood that the intervention was cost-effective was 66 % for both weight loss and QALYs gained.

The analysis in which QALYs were estimated according to the UK tariff also resulted in different outcomes. The ICUR in the phone group was €52,496, which was lower than in the main analysis (Table 5). The probability of cost-utility at €20,000/QALY was 8%. Similarly, the ICUR of the internet group was lower, €702 (Table 6). The probability of cost-utility was 71% at €20,000/QALY.

## Discussion

We set out to investigate the cost-effectiveness for weight reduction and cost-utility of a lifestyle program utilizing e-mail or phone counseling in comparison with self-help among overweight employees. Adherence to both interventions was limited. ICERs and ICURs implied that both interventions were more effective but also more costly than self help. However, the ICER and ICUR of the internet group were lower (respectively, €16/kg and €1337/QALY) than those of the phone group (€1009/kg and €245,243/QALY) and quite favorable. The phone group had the lowest probability of cost-effectiveness and cost-utility of all groups, whereas the internet group had the highest probability of cost-effectiveness at most willingness to pay thresholds, ranging from 47% at €0/kg to 80% at €450/kg, and 60% at €20,000/QALY. The sensitivity analyses generally confirmed the results from the main analysis, with some showing results that favored the internet group more than in the main analysis. The internet-based program therefore appears to be the preferred intervention.

Participants finished about half of the ten modules, with lower adherence in the internet group. The latter may be related to satisfaction with the different formats. At six months after baseline we conducted a process evaluation in which we asked the participants how satisfied they were with their group allocation: 91% of the phone group participants were satisfied compared with 78% of the internet group. The general appreciation, on a scale of 0 (lowest) to 10 (highest), was 7.4 for the phone format and 6.9 for the internet format.

In the main analyses we found no significant differences in body weight and QALYs gained, in comparison with the control group. Conversely, the complete case analysis showed significant weight loss in the internet group, and a trend towards significant weight loss in the phone group, compared with the control group. However, self-selection seems to have played a role in this result, judged by the differences in baseline and follow-up body weight between complete and incomplete cases. In addition, compared to the imputed cases, within-group weight loss in the complete cases of the internet group was similar, while weight loss decreased in the control group and increased in the phone group. This is surprising as we expected selection effects in the complete cases to result in higher within-group weight losses among all groups. The significant result among complete cases should be treated with caution.

Baseline health utility values were, on a scale from 0.00 (representing death) to 1.00 (representing perfect health), already high with values around 0.91. A problem of the EQ-5D utility index is that it does not discriminate between health statuses at the high end of the healthy utility range [34]. It is therefore not surprising that, in our relatively healthy population, differences in QALYs gained were small and not statistically significant. Research is going on to develop quality-of-life outcomes that are more sensitive to the immediate effects associated with preventive interventions [35].

When the UK tariff was applied, somewhat more QALYs were gained than with the NL tariff. Dutch respondents ascribe less weight than UK respondents to most dimensions on the EQ-5D [36]. This could mean that the UK-tariff is more sensitive to improvements in the EQ-5D dimensions than the NL tariff. Nevertheless, incremental gains remained small.

Health care costs in the internet group differed significantly from controls. Otherwise, no significant differences were found. Like most economic evaluations conducted alongside a RCT, our study was not powered to detect statistically significant differences in costs [37].

Results of the current study confirm those of two other studies that compared phone counseling of healthy adults on weight-related behaviors and concluded that it was not cost-effective compared with no intervention [12,13]. Both studies did not include societal costs nor had follow-up beyond the duration of the intervention. Regarding e-mail counseling interventions, no economic evaluations of these were identified. However, three trials found a combination of e-mail and phone counseling to be cost-effective in comparison usual care [9,10] or another intervention [11]. This suggests that a combination might be more cost-effective than the single interventions separately. Another explanation might lie in the methodological differences. First, conclusions in the three studies were based on complete cases (29% to 82% of all randomized participants) instead of imputed data sets, possibly leading to inflated effectiveness. Second, two of the studies [10,11] based their conclusion on the ICER but did not explore uncertainty around these outcomes [38]. Third, these studies did not include costs of productivity loss or all health care costs. Finally, all three studies reported post-intervention outcomes, as opposed to 18-months post-intervention in the current study. Weight rebound after initial weight loss is common, and was also seen in our sample [17,39].

The main purpose of the current economic evaluation was to identify which counseling mode produced the greatest amount of additional health at acceptable costs. It is not clear how much social decision makers (i.e., the Netherlands Ministry of Health, Welfare and Sport) are willing to pay for a kg of body weight lost. Furthermore, in the Netherlands, no maximum societal ceiling ratio per QALY gained is defined. A recent review commissioned by the Dutch government used a threshold of €20,000/QALY for preventive interventions [28], but higher thresholds have been proposed for both curative and preventive interventions, depending on the burden of disease [40]. Uncertainty about the cost-utility of the internet-based weight control program was appreciable, i.e. 40% at the €20,000/QALY threshold. The probability of its cost-effectiveness was a respectable 80% at €450/kg, but it seems unlikely that society is willing to pay this much. In addition, from the perspective of a Dutch company cost-effectiveness of this intervention was fairly uncertain, with a probability of 66% at zero WTP, for both QALYs and kg weight loss.

A limitation of this study is the rate of missing data. Missing data were multiply imputed for the main analysis. This method gives more valid results than complete case analysis and simple imputation methods such as baseline value carried forward [41,42]. Multiple imputation assumes that the available data are sufficient to predict missing costs and clinical outcomes, and that the costs and outcomes of those who provided data are similar to those who did not provide data. The latter assumption may not necessarily hold true, but cannot be tested. This makes it impossible to draw firm conclusions about the cost-effectiveness of the studied interventions.

Retention to the study is challenging in behavioral weight control studies. In the current study 45% of participants had dropped out after two years. Few previous studies in this field had a follow-up beyond one year. A modeling study estimated that 50% of participants in weight control studies will have dropped out after two years, which is comparable to the dropout we found [42]. This indicates that conclusions regarding efficacy and (cost-)effectiveness in the weight control field are seriously hampered. Future studies should prevent loss to follow-up. Upcoming technologies, like weighing scales that are connected to the internet, could make measurement of body weight for study-purposes less burdensome.[43] Research is needed to optimize cost diary and questionnaire design [44]. Finally, participants should be selected on motivation for continued participation in the trial [45] and motivation for completion of the study could be enhanced [46].

Another possible limitation of the study is that all cost data, except the costs of the intervention, were self-reported and that the cost diaries covered a relatively long period. More objective data, such as health claims data, are practically inaccessible in the Netherlands, so self-report of resource utilization is the common method. However, it is possible that participants completed the diaries retrospectively at the moment they had to return them instead of completing them prospectively. This could have resulted in a recall bias. Contradictory results on the influence of (period of) recall on the precision of self-reported sick leave and health care and medication use have been reported [47-50], but under-reporting of utilization seems likely. Nevertheless, we do not expect under-reporting to systematically differ between the intervention groups.

Strong points of the study are the randomized controlled design, the large study population of nearly 1400 participants, the relatively long follow-up period of two years, and the thorough presentation of uncertainty around the outcomes.

## Conclusions

The lifestyle program with phone counseling was not proven to be cost effective. The program with e-mail counseling showed some promising results but its cost-effectiveness was uncertain. Due to high loss to follow-up firm conclusions cannot be drawn. Future economic evaluations of weight control interventions should ensure that dropout is limited.

## Abbreviations

BMI: Body Mass Index; CE-plane: Cost-Effectiveness plane; CEAC: Cost-Effectiveness Acceptability Curve; EQ-5D: EuroQol-5D; ICER: Incremental Cost-Effectiveness Ratio; ICUR: Incremental Cost-Utility Ratio; QALY: Quality Adjusted Life Year; WTP: Willingness To Pay.

## Competing interests

Willem van Mechelen is director of Evalua Netherlands BV. All authors declare no competing interests.

## Authors’ contributions

WVM was the study supervisor and obtained the funding. MFVW and JCD implemented the trial and carried out recruitment and data collection. MWH produced the multiple datasets and gave statistical advice. JEB and MWVT advised on the estimation of costs and on statistics. MFVW performed all the analyses and wrote the paper. All authors contributed to the development of the study design and to reviewing, editing and approving the final version of the paper.

## Supplementary Material

Additional file 1 **File name: IJBNPA_ALIFE@Work_economic evaluation_additional file1.pdf.** Title: Calculation of intervention costs. Description: This file gives a detailed description of how the intervention costs were calculated. Click here for file

Additional file 2 **File name: IJBNPA_ALIFE@Work_economic evaluation_additional file2.pdf.** Title: CE-planes for the main analyses. Description: This file shows the CE-planes which were generated for the main analyses and that were not shown in the article. Click here for file
