# Supplementary material for: Economic evaluation of a weight control program with e-mail and telephone counseling among overweight employees: a randomized controlled trial
Source: Int J Behav Nutr Phys Act. 2012 Sep 11;9:112. doi: 10.1186/1479-5868-9-112 (PMC3499374; doi:10.1186/1479-5868-9-112)
Supplement: Additional file 1 — File name: IJBNPA_ALIFE@Work_economic evaluation_additional file1.pdf. Title: Calculation of intervention costs. Description: This file gives a detailed description of how the intervention costs were calculated. [file 1479-5868-9-112-S1.pdf]

Calculation of the intervention costs

Intervention costs were based on charges that were paid during development and implementation of the intervention. However, to calculate costs per participant, certain assumptions were made. The first assumption was that, when implemented under real life conditions, a counseling center would consist of twenty counselors, one supervisor and one general manager, and that the center would be able to counsel 3000 individuals per year. Thus, the fixed costs of the development and implementation of the program were spread over 1500 participants per counseling mode (phone and internet) yearly. Additionally, we assumed the program to be depreciated over a period of five years. Intervention costs were calculated to a fixed (annual) price and a variable price per participant. The fixed price of the phone intervention consisted of costs for the development of the phone materials (project management, content development and graphic design), print costs for materials, step counter costs and counseling costs (office furniture and equipment, supervision and management), at a total of €69 per participant (Table 1). The fixed price of the internet intervention consisted of costs for the development of the website (project management, content development, graphic design and programming), costs for technical maintenance of the website and hosting, text messaging costs, print costs for materials and counseling costs (office furniture and equipment, supervision and management), at a total of €65 per participant (Table 1). For both the phone and internet intervention a variable cost was computed per participant, based on the amount of counseling actually received. After each counseling session, counselors recorded the content of the counseling session and the total time that the session had taken, including administration. After the intervention had ended, total recorded time

was compared with available time based on work contracts. This showed that counselors spent 32% of the available time on counseling. As very little time was spent on overhead, such as meetings, and a decrease in counseling contacts over time not fully accounted for the discrepancy in available time and recorded time, we concluded that time spent counseling was underreported. However, we do not know the actual amount of underreporting. We therefore decided to ascribe all personnel costs for counseling to the interventions. Considering that under real-world conditions the intervention price should cover all personnel costs and not just time spent counseling, this seems a fair decision. Counseling costs per minute as based on employment costs were multiplied by 100/31.8 = 3.14, resulting in a cost of €1.14 per recorded minute. The employment costs of the counselors included wages, employers' contribution to social taxes, insurance and schemes, and 16% overhead. This overhead accounted for use of offices, central heating, general personnel and postal facilities. Costs of phone calls were also included in this overhead; this is why these were not separately included in the overall costs of the phone counseling sessions.

The total intervention costs per participant were estimated by adding the fixed and variable costs (Table 1).

Table 1 Price of the intervention

		Costs in € in 2004
Phone intervention, fixed costs		
Consisting of:		
Development	€1.74	
Materials	€23.80	
Management/supervision	€43.46	68.99
Internet intervention, fixed costs		
Consisting of:		
Development	€6.22	
Website maintenance	€1.68	
Hosting	€2.52	
Text messaging	€3.93	
Materials	€6.68	
Management/supervision	€43.46	64.67
Counseling, variable costs (per recorded minute)		1.14
