# Supplementary material for: Economic evaluation of a weight control program with e-mail and telephone counseling among overweight employees: a randomized controlled trial
Source: Int J Behav Nutr Phys Act. 2012 Sep 11;9:112. doi: 10.1186/1479-5868-9-112 (PMC3499374; doi:10.1186/1479-5868-9-112)

CE-plane phone vs control for body weight

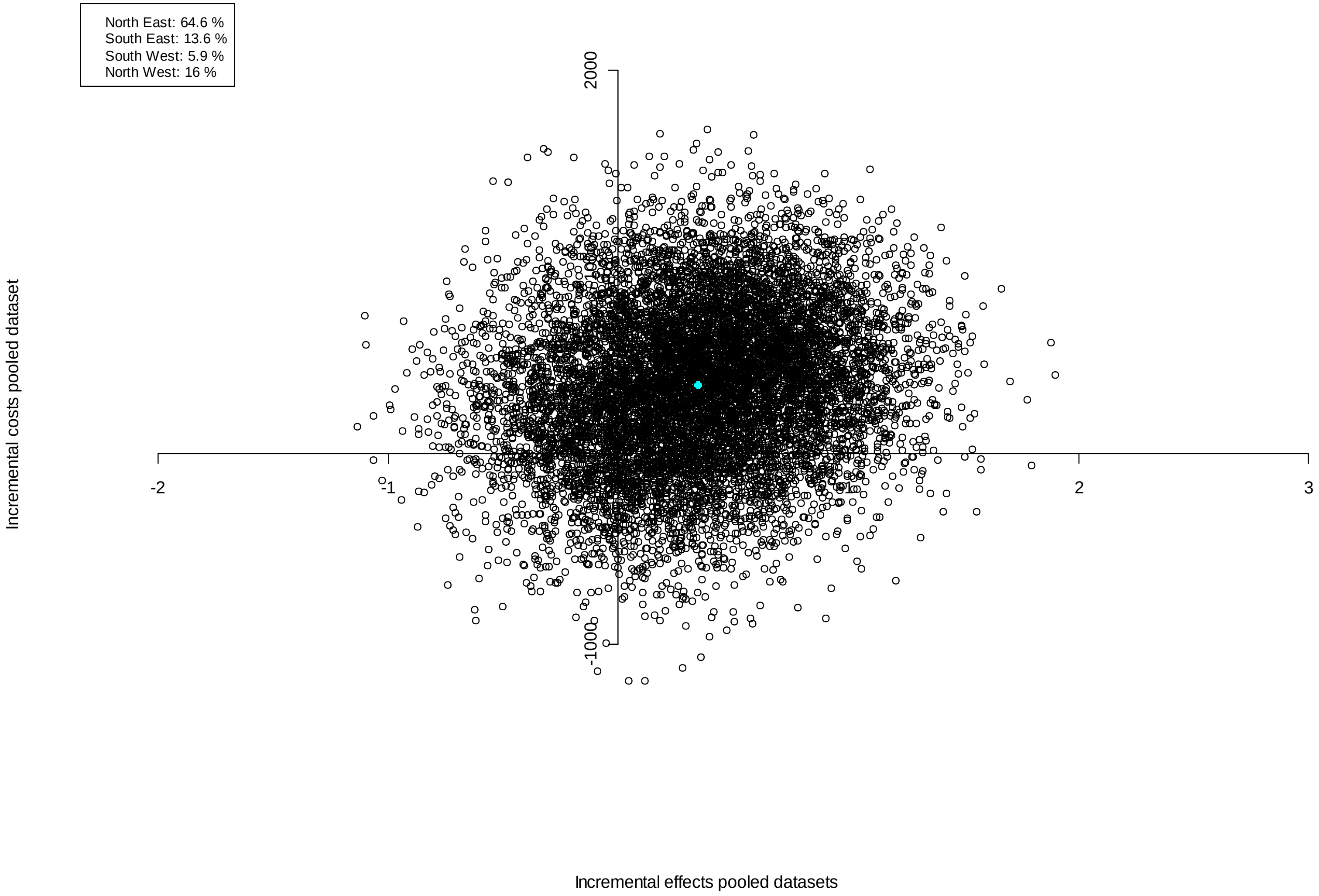

CE-plane internet vs control for body weight

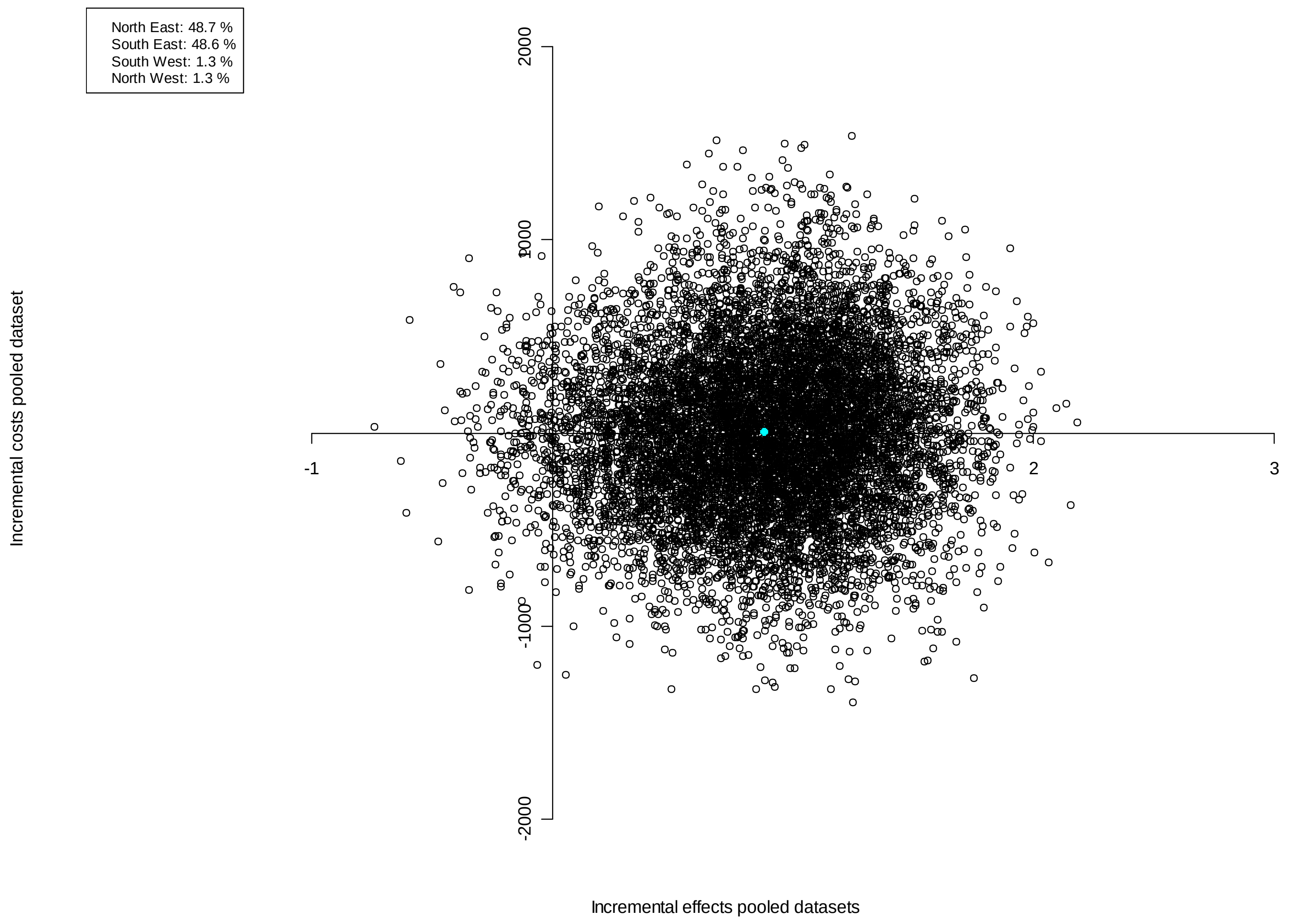

Cost utility-plane phone vs control for QALYs gained

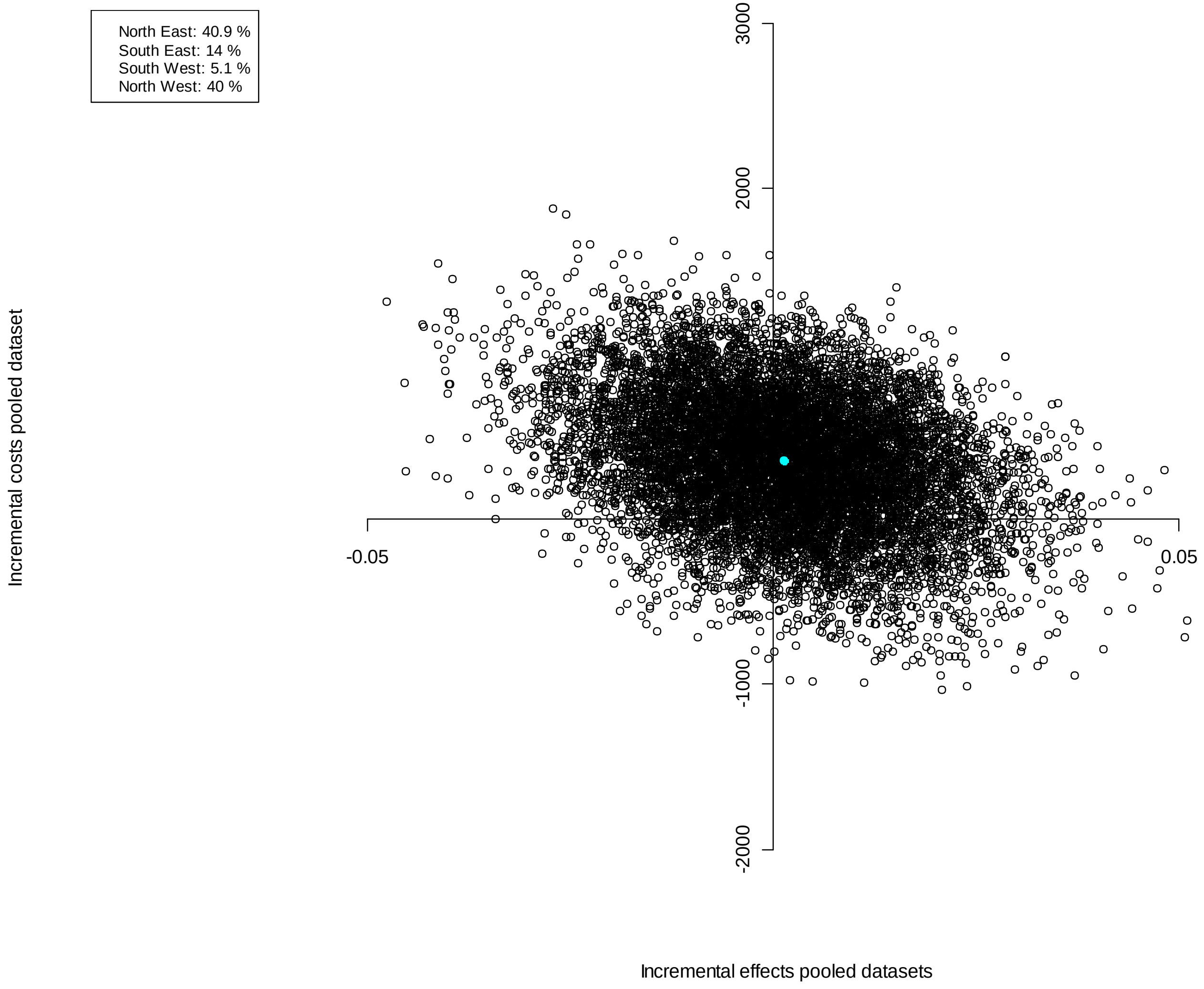

Cost utility-plane internet vs control for QALYs gained

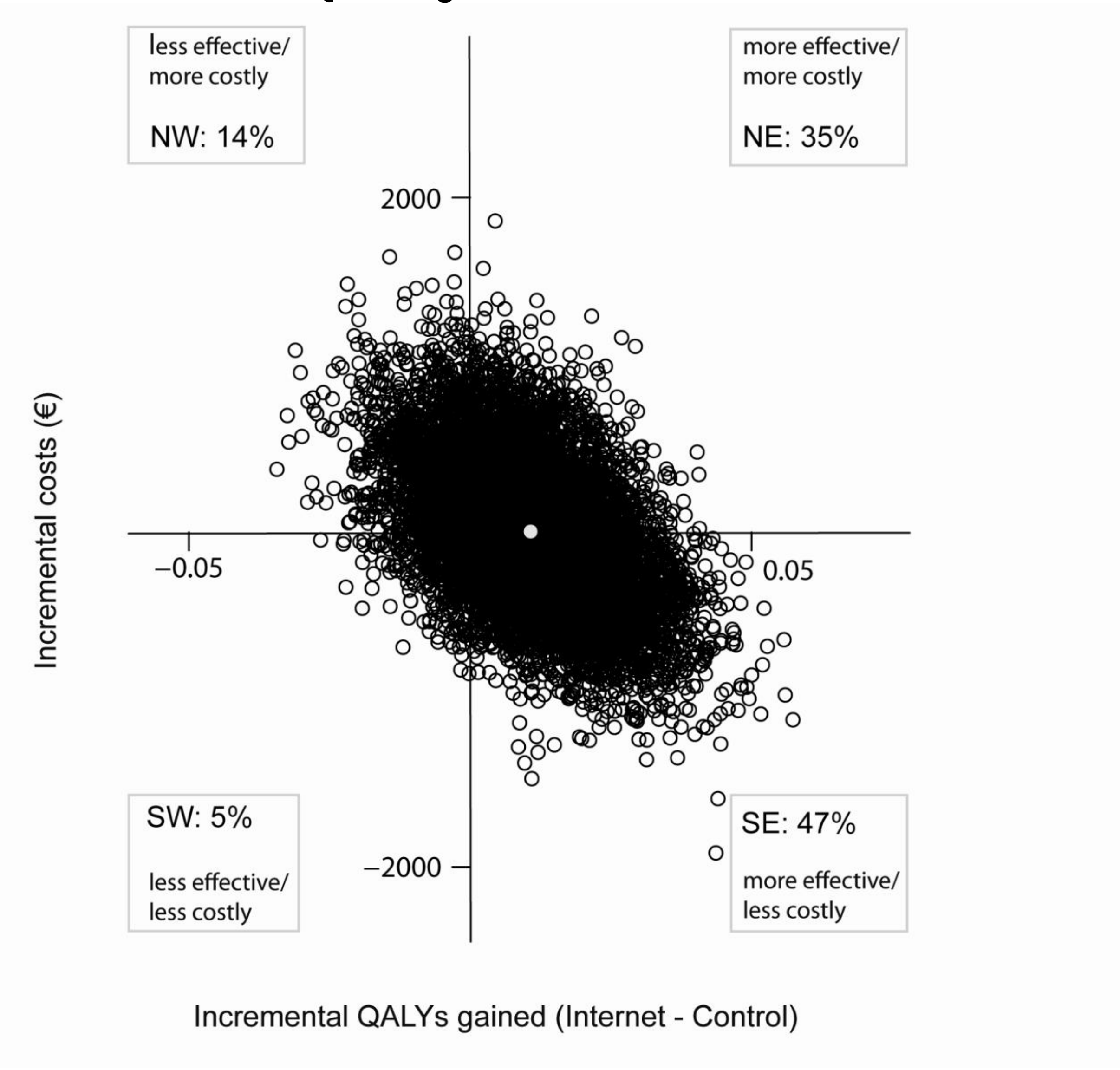

Supplement: Additional file 2 — File name: IJBNPA_ALIFE@Work_economic evaluation_additional file2.pdf. Title: CE-planes for the main analyses. Description: This file shows the CE-planes which were generated for the main analyses and that were not shown in the article. [file 1479-5868-9-112-S2.pdf]
